# *GnIH* Modulates Proliferation, Apoptosis, and Steroid Hormone Secretion in Tibetan Sheep Granulosa Cells

**DOI:** 10.3390/ani16213517

**Published:** 2026-11-06

**Authors:** Yuxiang Zhang, Litan Zhang, Xinyu Ji, Zelin Liu, Bin Huang, Na He, Xin Gu, Yuting Deng, Junxia Zhang

**Affiliations:** 1 College of Agriculture and Animal Husbandry, Qinghai University, Xining 810016, China; ys240951330642@mailbox.qhu.edu.cn (Y.Z.);; 2 Qinghai Provincial Sheep Breeding and Promotion Service Center, Haibei 812200, China; 3 Tongde County Animal Husbandry and Veterinary Station, Tongde 813200, China

**Keywords:** Tibetan sheep, *GnIH*, ovarian granulosa cells (GCs), proliferation, apoptosis, steroid hormone secretion

## Abstract

Gonadotropin-inhibitory hormone (*GnIH*) is a key neuropeptide that negatively regulates animal reproduction. This study aims to elucidate its functional mechanism in Tibetan sheep, a characteristic plateau-adapted breed. A total of 12 Tibetan sheep were used in this study. For granulosa cell experiments, granulosa cells were isolated from the ovaries of three independent Tibetan sheep to generate three biological replicates, and three technical replicates were set for qPCR and Western blot detection within each biological replicate. The mRNA levels of *GnIH* in gonadal axis tissues, including the hypothalamus, pituitary, ovary, uterus and oviduct, were detected using RT-qPCR, and fluorescence in situ hybridization was performed to identify the subcellular localization of *GnIH* in ovarian granulosa cells. The overexpression plasmid pcDNA3.1 *GnIH* and *GnIH*-targeted siRNA fragments were constructed and transfected into Tibetan sheep ovarian granulosa cells. Cell proliferation, apoptosis, cell cycle distribution, and hormone secretion were examined with the aid of CCK 8 assay, flow cytometry, RT-qPCR and ELISA. The results showed that *GnIH* was widely expressed in gonadal axis tissues of Tibetan ewes. The highest mRNA abundance of *GnIH* was found in the hypothalamus, followed by the ovary. *GnIH* signals were mainly localized in the cytoplasm of ovarian granulosa cells. *GnIH* overexpression reduced granulosa cell viability, arrested cell cycle progression, upregulated proapoptotic genes *BAX, Caspase 3* and *Caspase 9*, and downregulated the proliferation-related gene *PCNA*. Simultaneously, the secretion of estradiol (E_2_) and progesterone (P_4_) was simultaneously reduced. Endogenous *GnIH* knockdown enhanced the proliferative capacity of granulosa cells, inhibited cell apoptosis, uplifted the proportions of S phase and G2/M phase cells, and elevated estradiol secretion. These results confirm that *GnIH* acts as a negative regulator of granulosa cell function. It inhibits follicular development by suppressing cell proliferation, promoting cell apoptosis and reducing steroid hormone synthesis. These in vitro observations offer preliminary clues regarding the regulatory role of *GnIH* in granulosa cell proliferation and apoptosis.

## Simple Summary

Tibetan sheep are important livestock adapted to the harsh high-plateau environment; therefore, improving their reproductive performance is of great significance for local animal husbandry. Gonadotropin-inhibitory hormone (*GnIH*, also known as RFRP-3 in mammals) is a neuropeptide that suppresses animal reproduction, but its function in Tibetan sheep ovarian granulosa cells remains unclear. In this study, we detected the distribution of the *GnIH* gene in key tissues of the gonadal axis of female Tibetan sheep and performed functional verification using ovarian granulosa cells. The results showed that the GnIH gene was widely expressed in reproductive tissues of Tibetan sheep, with the highest mRNA abundance in the hypothalamus followed by the ovary, and its signal was mainly localized in the cytoplasm of granulosa cells. Overexpression of *GnIH* inhibited granulosa cell proliferation, promoted apoptosis, arrested the cell cycle, and reduced the secretion of estradiol and progesterone. Conversely, *GnIH* knockdown facilitated granulosa cell proliferation, suppressed apoptosis, and increased estradiol secretion. This study confirms that *GnIH* negatively regulates granulosa cell proliferation and inhibits follicular development. These findings help decipher the reproductive regulatory mechanism of plateau Tibetan sheep and provide a theoretical basis for Tibetan sheep breeding research.

## 1. Introduction

Tibetan sheep are highly adapted indigenous livestock breeds distributed in the alpine pastoral regions of the Qinghai–Tibet Plateau. Following long-term domestication under plateau conditions [1], they have evolved unique physiological characteristics to cope with extreme habitats featuring high altitude, low temperature, hypoxia and seasonal forage scarcity. Together with yaks and Tibetan goats, Tibetan sheep represent one of the few domesticated animal species capable of surviving and reproducing robustly under harsh plateau environments and serve as core genetic resources supporting animal husbandry on the Qinghai–Tibet Plateau [2]. Nevertheless, long-term adaptive responses to high-altitude stress have profoundly reshaped their reproductive traits and resulted in a generally low reproductive efficiency, which severely restricts the sustainable development of the characteristic Tibetan sheep industry [3].

Sheep reproductive performance is regulated by three core physiological processes of follicular development, ovulation, and steroid hormone secretion. As the principal functional cells within ovarian follicles, granulosa cells (GCs) exert decisive effects on sheep reproduction throughout follicle growth and maturation [4]. GCs establish bidirectional signal communication with oocytes to modulate follicular cell proliferation and differentiation and are responsible for synthesizing and secreting key reproductive hormones, including estradiol and progesterone. Dysfunctional granulosa cells directly trigger follicular atresia, reduce ovulation number, and ultimately decrease fecundity in female animals [5]. Accordingly, ovarian granulosa cells are an essential cellular model for deciphering molecular regulatory mechanisms underlying livestock reproduction. Nevertheless, the biological functions underlying GC growth and development, as well as the molecular mechanisms by which GCs modulate oocyte maturation and follicular development, remain completely unelucidated. Further exploration of novel regulatory factors is required to fully clarify the roles of GCs in follicular development, such as gonadotropin-inhibitory hormone (GnIH). As a member of the *LPXRFamide* (Rfamide) neuropeptide family, *GnIH* is an evolutionarily conserved negative regulator of reproduction across vertebrates [6]. Moreover, it not only suppresses the synthesis and release of gonadotropins via the hypothalamic–pituitary–gonadal (HPG) axis but also directly acts on ovarian granulosa cells through autocrine and paracrine pathways [7]. Existing evidence demonstrates that *GnIH* inhibits granulosa cell proliferation, induces cell apoptosis, downregulates the expression of steroidogenic genes such as CYP19A1 and StAR, reduces the secretion of estradiol and progesterone, and blocks follicular development at the cellular level [8,9,10]. Consistently, *GnIH* has been verified to persistently and negatively regulate follicular development, ovulation, and corpus luteum formation by restraining granulosa cell proliferation, promoting apoptosis, and inhibiting steroidogenesis, and these biological effects have been widely validated in granulosa cells of mice, poultry, and domestic mammals [11,12,13,14,15].

Although the inhibitory effects of *GnIH* on follicular development and reproductive function have been well documented in conventional sheep, goats, cattle, mice, and other model animals [16,17,18], granulosa cell proliferation, differentiation, steroidogenesis, and crosstalk with oocytes have been extensively studied across a range of domestic animal species. Despite this solid foundational knowledge, the specific roles of *GnIH* in regulating proliferation and apoptosis of Tibetan sheep ovarian granulosa cells remain unclear. Systematic studies regarding the expression and localization of *GnIH* in the HPG axis of Tibetan sheep remain unreported in the literature; furthermore, few studies have visualized its subcellular localization in ovarian granulosa cells or validated its local functions via combined overexpression and siRNA-mediated knockdown assays using primary granulosa cells from this breed. Furthermore, there is still a lack of comprehensive functional validation through overexpression and RNA interference in primary granulosa cells. Accordingly, the present study attempts to fill this knowledge gap by investigating the expression and distribution of *GnIH* in HPG axis tissues of Tibetan sheep. Combined with in vitro overexpression and interference assays in granulosa cells, we systematically elucidated the regulatory mechanisms of the *GnIH* genes on granulosa cell proliferation, cell cycle, apoptosis, and steroid hormone secretion in Tibetan sheep. This in-depth study aims to yield new insights into the molecular regulatory mechanisms of *GnIH* in regulating GC function and offer a reference for improving the reproductive performance of Tibetan sheep.

## 2. Materials and Methods

### 2.1. Experimental Animals and Sample Collection

A total of 12 multiparous Tibetan ewes (parity = 2, 24 months old) at the proestrous stage, non-pregnant, and under identical feeding, immunization, and management conditions were selected from Jinhe Breeding Cooperative, Gonghe County, Hainan Tibetan Autonomous Prefecture, Qinghai Province, for this research. All sheep were slaughtered by professional technicians from the Gonghe County Animal Husbandry Department. After exsanguination, hypothalamus, pituitary, uterus, oviduct, and ovary tissues were collected from six ewes, immediately frozen in liquid nitrogen and transported to the laboratory, followed by long-term storage at −80 °C for total RNA extraction. Of the remaining six ewes, ovaries were collected and preserved in phosphate-buffered saline (PBS(Cat. No. BA4041, Baso Biotechnology Co., Ltd., Zhuhai, Guangdong, China)) supplemented with penicillin and streptomycin. Ovaries from three ewes were used for in situ hybridization and hematoxylin–eosin (HE) staining, whereas ovaries from the other three ewes were used to isolate granulosa cells from healthy 2–6 mm antral follicles for subsequent cell culture. For granulosa cell functional assays, granulosa cells were isolated from ovaries of three independent ewes to establish three biological replicates; three technical replicates were prepared for qPCR and Western blot analysis within each biological replicate. All animal tissue handling procedures adhered closely to the Animal Ethics Standards of Qinghai University (Approval No. PJ202501-119).

### 2.2. Hematoxylin and Eosin (HE) Staining

Ovarian cortical tissues from Tibetan ewes were embedded in paraffin and serially sectioned at 5 μm. The sections underwent routine histological processing: deparaffinization, hematoxylin–eosin (HE) staining, dehydration, clearing, and mounting. Histological observation and image acquisition were performed using an optical microscope.

### 2.3. In Situ Hybridization

Fresh ovarian tissues of the Tibetan ewes were fixed in DEPC-treated fixative for more than 12 h, followed by gradient ethanol (Cat. No. 100092683, Sinopharm Chemical Reagent Co., Ltd., Shanghai, China) dehydration and paraffin embedding. Paraffin sections were baked at 62 °C for 2 h and sequentially deparaffinized with xylene(Cat. No. 10023418, Sinopharm Chemical Reagent Co., Ltd., Shanghai, China) to distilled water. After heat-induced antigen retrieval, sections were permeabilized via proteinase K(Cat. No. 1245680100, Solarbio Science & Technology Co., Ltd., Beijing, China) digestion. The target oligonucleotide probe was labeled with CY3, and a scrambled probe without complementary sequence was used as the negative control to exclude non-specific binding. Prehybridization was conducted at 37 °C for 1 h, and then the prehybridization solution was replaced with probe hybridization solution for overnight incubation at a constant temperature. Gradient SSC (Cat. No. S1030, Solarbio Science & Technology Co., Ltd., Beijing, China) washing was performed to eliminate non-specific binding signals. Nuclei were counterstained with DAPI(Cat. No. C02-04002, Beijing Biosynthesis Biotechnology Co., Ltd., Beijing, China) in the darkness, and sections were mounted with an anti-fluorescence quenching medium. Fluorescence images were captured using a fluorescence microscope. Three random fields of view were selected for each section to assess signal localization and relative fluorescence intensity. Tissue expression data were analyzed independently for each individual ewe. The localization and relative abundance of target nucleic acids (mRNA/miRNA/lncRNA) were determined according to blue DAPI nuclear signals, green FAM fluorescence, and red CY3 fluorescence.

### 2.4. Isolation and Culture of Ovarian Granulosa Cells

Fresh, untreated ovaries were rinsed in sterile PBS (Cat. No. BA4041, Baso Biotechnology Co., Ltd., Zhuhai, Guangdong, China). Only healthy antral follicles with diameters of 2–6 mm were selected for granulosa cell isolation. Approximately 15–20 follicles were collected from each ovary, and ovaries from one ewe were used for each independent cell preparation. Surface follicles were incised with a sterile scalpel, and follicular fluid was allowed to drain into pre-equilibrated collection medium. The cell suspension was centrifuged at 2000 rpm for 5 min. The supernatant was discarded, leaving a white cell pellet. The pellet was resuspended in 2 mL culture medium containing antibiotics (1% penicillin–streptomycin; 100 U/mL penicillin and 100 μg/mL streptomycin), then centrifuged at 1000 rpm for 5 min; this wash was repeated three times to obtain a granulosa-cell pellet. The basal culture medium was DMEM/F12 supplemented with 10% fetal bovine serum (FBS, Cat. No. 10091148, Gibco, Grand Island, NY, USA) and 1% penicillin–streptomycin solution (100 U/mL penicillin and 100 μg/mL streptomycin). Granulosa cells were seeded at a density of 1 × 10^6^ cells/mL in 6-well plates and cultured at 37 °C under 5% CO_2_ and saturated humidity. Cell identity was confirmed by the standardized follicle dissection procedure and the typical cobblestone-like morphology observed under an inverted microscope (Model Ts2R-FL, Nikon, Tokyo, Japan), which is characteristic of ovarian granulosa cells. All functional assays were performed using primary granulosa cells without passaging.

### 2.5. Plasmid Construction

The coding sequence of Tibetan sheep *GnIH* was retrieved from the NCBI database (National Center for Biotechnology Information, Bethesda, MD, USA; NCBI Reference Sequence: NM_001127268.1). The pcDNA3.1 vector was adopted to construct the *GnIH* overexpression plasmid, which was synthesized by General Biosystems (Anhui, China) Co., Ltd. (Chuzhou, China). Three specific siRNA sequences targeting *GnIH* and one negative control siRNA were also synthesized by the same company and are listed in Table 1.

### 2.6. Cell Transfection

When cell confluence reached 60–70%, transfection was performed following the manufacturer’s instructions of Lipofectamine 3000 (Invitrogen, Carlsbad, CA, USA). Granulosa cells were seeded at 1 × 10^6^ cells per well in 6-well plates, with a final transfection volume of 2 mL per well. Plasmid or siRNA was diluted with the Opti-MEM (Gibco, Grand Island, NY, USA) medium and mixed with Lipofectamine 3000 reagent. For plasmid transfection, 2 μg of pcDNA3.1-GnIH plasmid DNA and 4 μL of Lipofectamine 3000 were used per well. For siRNA transfection, siRNA was used at a final concentration of 50 nM, with 4 μL of Lipofectamine 3000 per well. After 10~15 min of incubation at room temperature, the transfection complexes were added to cells. The medium was refreshed 4~6 h post-transfection, and cells were harvested at 24~48 h for downstream experiments. Cells were harvested at different post-transfection time points according to the type of assay: 24 h for cell apoptosis assay and cell cycle detection; 48 h for qPCR amplification, Western blot analysis and steroid hormone secretion detection.

### 2.7. RT-qPCR Experiment

#### 2.7.1. Total RNA Extraction

Total RNA of samples was extracted using TRNzol reagent. After lysis at room temperature for 10 min, 200 µL of chloroform (Cat. No. 10006818, Sinopharm Chemical Reagent Co., Ltd., Shanghai, China) was added, followed by manual shaking and mixing, standing for 10 min, and centrifugation at 12,000 rpm and 4 °C for 15 min. The upper aqueous phase containing RNA was collected, mixed with an equal volume of pre-cooled isopropanol (Cat. No. 80109218, Sinopharm Chemical Reagent Co., Ltd., Shanghai, China) and precipitated at −20 °C for 15 min. After centrifugation at the same rotating speed and temperature for 10 min to harvest the RNA precipitate, the precipitate was washed twice with pre-cooled 75% ethanol (Cat. No. 10009218, Sinopharm Chemical Reagent Co., Ltd., Shanghai, China) dissolved in DEPC water (Cat. No. RT121-02, TIANGEN BIOTECH (BEIJING) CO., LTD., Beijing, China) after drying. RNA concentration and purity were detected by NanoDrop 2000 (Thermo Fisher Scientific, Wilmington, DE, USA). RNA integrity was assessed by 1.5% agarose gel electrophoresis using a PowerPac 300 electrophoresis power supply (Bio‑Rad Laboratories, Hercules, CA, USA). Qualified RNA was preserved at −80 °C.

#### 2.7.2. Reverse Transcription

A total of 5 μg of intact RNA was transferred to an RNase‑free PCR tube, and a 20 μL RNase‑free reverse transcription reaction system was assembled on ice using the RevertAid First Strand cDNA Synthesis Kit (Cat. No. K1622, Thermo Fisher Scientific, Waltham, MA, USA): 4 µL 5× Reaction Buffer, 1 µL reverse transcription primer, 5 µg RNA, 1 µL Ribolock RNase Inhibitor (20 U/µL), 1 µL RevertAid M‑MuLV Reverse Transcriptase (200 U/µL), 2 µL 10 mM dNTP Mix, and RNase/DNase‑free ddH_2_O (Cat. No. RT121‑02, TIANGEN BIOTECH (BEIJING) CO., LTD., Beijing, China) to a final volume of 20 μL. The mixture was gently mixed and briefly centrifuged. When gene‑specific primers (Genscript, Nanjing, Jiangsu, China) were used, the reaction was incubated at 42 °C for 60 min. For random hexamer primers, incubation was performed at 25 °C for 5 min, followed by 60 min at 42 °C. For RNA templates with high GC content or stable secondary structures, the template was combined with the primer mixture, briefly centrifuged, incubated at 65 °C for 5 min, chilled on ice, centrifuged again, and maintained on ice before setting up the reaction. The reverse transcription reaction was terminated by heating at 70 °C for 5 min. The resulting cDNA was either diluted immediately for qPCR or stored at −20 °C for short‑term preservation.

#### 2.7.3. qPCR Amplification

Gene expression levels in Tibetan sheep granulosa cells were quantified using a StepOnePlus real-time PCR instrument (Life Technologies, Wilmington, DE, USA). Detailed primer information is listed in Table 2. Diluted cDNA (approximately 200 ng/µL) served as the PCR template. The PCR reaction system was prepared as follows: 1 µL cDNA (200 ng/µL), 0.5 µL forward primer (10 µM), 0.5 µL reverse primer (10 µM), 10 µL SYBR mix, 0.4 µL ROX Reference Dye (50×), and Rnase/Dnase-free ddH_2_O to a final volume of 20 µL. The amplification program consisted of an initial denaturation step at 94 °C for 30 s, followed by 40 cycles of 94 °C for 5 s and 60 °C for 30 s. Melting curve analysis was conducted after amplification: 95 °C for 15 s, 60 °C for 1 min, with a gradual temperature increase of 0.3 °C every 15 s up to 95 °C, and a final hold at 95 °C for 15 s. Primer specificity was confirmed by melting curve profiles; a single distinct peak indicated specific amplification. Amplification efficiency for each primer pair was determined from standard curves constructed with serially diluted cDNA, and only primer pairs with amplification efficiency ranging from 90% to 110% were used. Ct values were acquired using ABI StepOnePlus software, and each sample was run in triplicate. Β-actin was selected as the reference gene for normalization, and the relative fold change in gene expression was calculated using the 2^−∆∆CT^ method. It is a commonly used housekeeping gene in ovine granulosa cell research. Our preliminary tests showed that its expression was stable among all treatment groups, consistent with previous studies on ruminant granulosa cells.

### 2.8. Western Blot Assay

#### 2.8.1. Protein Extraction

Total protein was extracted from transfected granulosa cells using RIPA lysis buffer containing phenylmethylsulfonyl fluoride (PMSF) at a final concentration of 1 mM. Briefly, the culture medium was aspirated, and cells were washed twice with pre-chilled PBS. For cells cultured in a single well of a 6-well plate, 150 μL of RIPA lysis buffer was added. Samples were thoroughly lysed on ice for 30 min and then centrifuged at 12,000 rpm and 4 °C for 10 min. The resulting supernatant was collected. Protein concentration was quantified via the BCA assay, and suitable protein aliquots were stored at −80 °C for downstream analysis.

#### 2.8.2. Western Blot

In total, 80 μg of total protein was mixed with 5× protein loading buffer and denatured by boiling for 10 min. Protein separation was performed by SDS-PAGE electrophoresis at a constant voltage of 140 V (Bio‑Rad Laboratories, Hercules, CA, USA). After electrophoresis, the gel and methanol-activated PVDF membranes (0.45 µm, Cat. No. IPVH00010, Merck KGaA, Darmstadt, Germany) were equilibrated in transfer buffer for 20 min, followed by transmembrane electrophoresis at a constant current of 200 mA for 1 h. The membranes were blocked with 5% skim milk (Cat. No. P0216-1500g, Beyotime Biotechnology, Shanghai, China) at room temperature. For *GnIH*, the primary antibody was rabbit anti-RFRP3 (Bioss, Cat. No. bs-19339R, diluted at 1:500); the internal reference anti-GAPDH antibody was mouse anti-GAPDH (Proteintech, Cat. No. 60004-1-Ig, diluted at 1:2000) and was used as the loading control for blots. Primary antibodies were diluted at a ratio of 1:1000 and incubated with the membranes overnight at 4 °C. After thorough washing with TBST buffer (ZK-Bioscience, Tianjin, China), the corresponding secondary antibodies were HRP-conjugated goat anti-rabbit IgG (Proteintech, Cat. No. RGAR001, 1:4000) and HRP-conjugated goat anti-mouse IgG (Proteintech, Cat. No. RGAM001, 1:4000). The secondary antibodies (1:3000 dilution) were incubated at room temperature for 1 h. Following another round of TBST washing, ECL chemiluminescence substrate (Cat. No. BL520B, Biosharp, Hefei, Anhui, China) was evenly applied, and the membranes were incubated in the darkness for 2 min. Protein bands were visualized using a chemiluminescence imaging system (Shanghai Tanon Technology Co., Ltd., Shanghai, China), and the relative gray intensity of each band was quantitatively analyzed using Gelpro32 software (Media Cybernetics, Rockville, MD, USA). Band intensity was quantified using Gelpro32 software based on three independent biological replicates. The relative expression level of the target protein was normalized to GAPDH. Statistical analysis was performed to compare differences between groups.

### 2.9. Cell Proliferation Assay

The proliferative capacity of ovarian granulosa cells after *GnIH* overexpression and knockdown was assessed using the Cell Counting Kit-8 (CCK-8) assay (Baiotai Biotechnology, Shanghai, China). Briefly, granulosa cells from the negative control and experimental groups were cultured at 37 °C under 5% CO_2_ for 12 h, 24 h, 48 h and 72 h following transfection. The CCK-8 working solution was then added following the manufacturer’s instructions. The optical density (OD) at 450 nm was measured with a microplate reader (Thermo Fisher Scientific, Waltham, MA, USA) to indicate dynamic changes in cell viability.

### 2.10. Cell Apoptosis Assay

At 24 h post‑transfection, cells were harvested, washed, and counted. Cells were double‑stained using the Annexin V‑FITC apoptosis detection kit (Cat. No. C1062, Beyotime Biotechnology, Shanghai, China), incubated in the dark, and cell apoptosis was detected using a Novocyte 2060R flow cytometer (Agilent Technologies, Santa Clara, CA, USA).

### 2.11. Cell Cycle Detection

At 24 h post-transfection, cell cycle distribution was detected by flow cytometry with propidium iodide (PI) staining. Briefly, after the treatment of transfected granulosa cells, the culture supernatant was collected. Adherent cells were washed twice with PBS and digested with trypsin. The collected supernatant was added to terminate digestion, and all cells were harvested. Cells were centrifuged at 1000× *g* for 3–5 min, the supernatant was discarded, and the cell pellet was resuspended in pre-chilled PBS. After another centrifugation step, cells were fixed in pre-cooled 70% ethanol at 4 °C overnight. Before staining, cells were washed twice with ice-cold PBS to remove ethanol. Then 0.5 mL of PI staining solution containing Rnase A was added to resuspend the cells, followed by incubation at 37 °C in the dark for 30 min. The red fluorescence signal was detected on the PI channel of a flow cytometer, and DNA content was analyzed to determine the proportion of cells in G0/G1, S and G2/M phases. Three independent biological replicates were performed.

### 2.12. Steroid Hormone Secretion Assay

Cell culture supernatants were harvested at 48 h post‑transfection and clarified by brief centrifugation. Concentrations of estradiol and progesterone were quantified using the Estrogen (E_2_) ELISA kit (Cat. No. JL54813, Jianglai Biotechnology, Shanghai, China, 48 tests) and Ovine progesterone (P_4_) ELISA kit (Cat. No. PE-81025O2, Puxi Biotechnology Co., Ltd., Shanghai, China, 48 tests), respectively, following the manufacturer’s instructions. To eliminate confounding effects caused by differences in viable cell density among groups, the measured hormone levels were normalized to the total protein content of granulosa cells harvested from the same well. Total protein was determined using a BCA protein assay kit (Cat. No. PB1146W5000T, Puxi Biotechnology Co., Ltd., China, 5000 tests). Progesterone (P_4_) and estradiol (E_2_) concentrations are expressed as pg/mL. All samples were assayed in triplicate, and the results were compared with the corresponding control group.

### 2.13. Statistical Analysis

Statistical analysis was performed using SPSS 26.0 software. Student’s *t*-test was adopted for comparisons between two groups, and one-way analysis of variance (one-way ANOVA) followed by Tukey’s multiple comparison test was used when three or more groups were compared. The longitudinal data of cell proliferation activity and hormonal secretion following *GnIH* overexpression and knockdown were analyzed using repeated-measures ANOVA. All experimental data were presented as mean ± standard error of the mean (SEM). Figures were generated using GraphPad Prism 9.0 where appropriate. * *p* < 0.05 and ** *p* < 0.01 indicate statistically significant differences between groups.

## 3. Results

### 3.1. Expression of GnIH Genes in the Tissues of Tibetan Sheep

**Figure 1:**
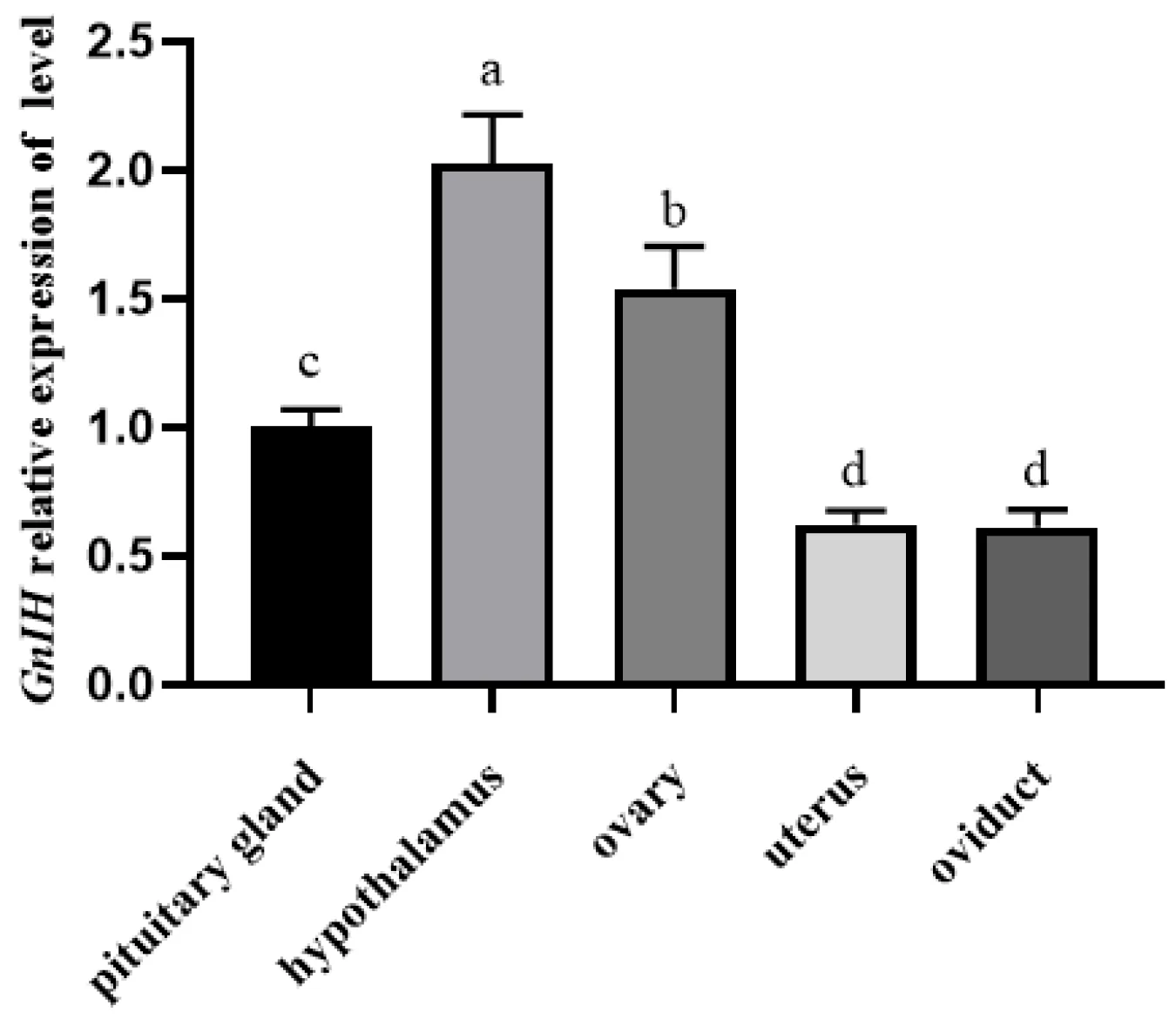
mRNA expression of *GnIH* genes in different gonadal axis tissues. Different lowercase letters above the bars indicate significant differences at *p* < 0.05, but insignificant differences at *p* > 0.05.

Shown in Figure 1 are the mRNA levels of *GnIH* genes in hypothalamus, pituitary, uterus, ovary and oviduct tissues of the Tibetan sheep. The results indicated that *GnIH* mRNA exhibited the highest expression in the hypothalamus (*p* < 0.05). The expression level of *GnIH* in the ovary was significantly higher than that in the pituitary, uterus and oviduct (*p* < 0.05). Similarly, pituitary *GnIH* mRNA abundance was markedly elevated relative to that of the uterus and oviduct (*p* < 0.05), but no significant difference existed between the uterus and the oviduct (*p* > 0.05).

### 3.2. Expression and Localization of GnIH Genes in the GCs of Tibetan Sheep

**Figure 2:**
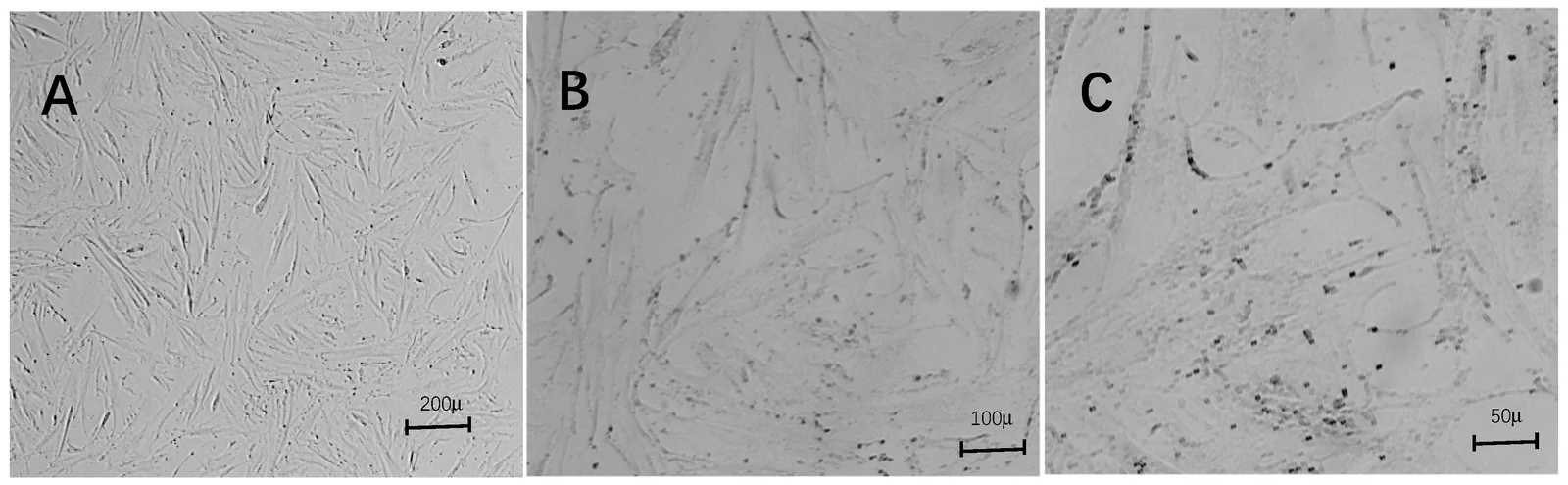
Growth morphology of in vitro cultured ovarian granulosa cells from Tibetan sheep. Panels (**A**–**C**) show views at different magnifications: (**A**) 40×; (**B**) 100×; (**C**) 200×.

**Figure 3:**
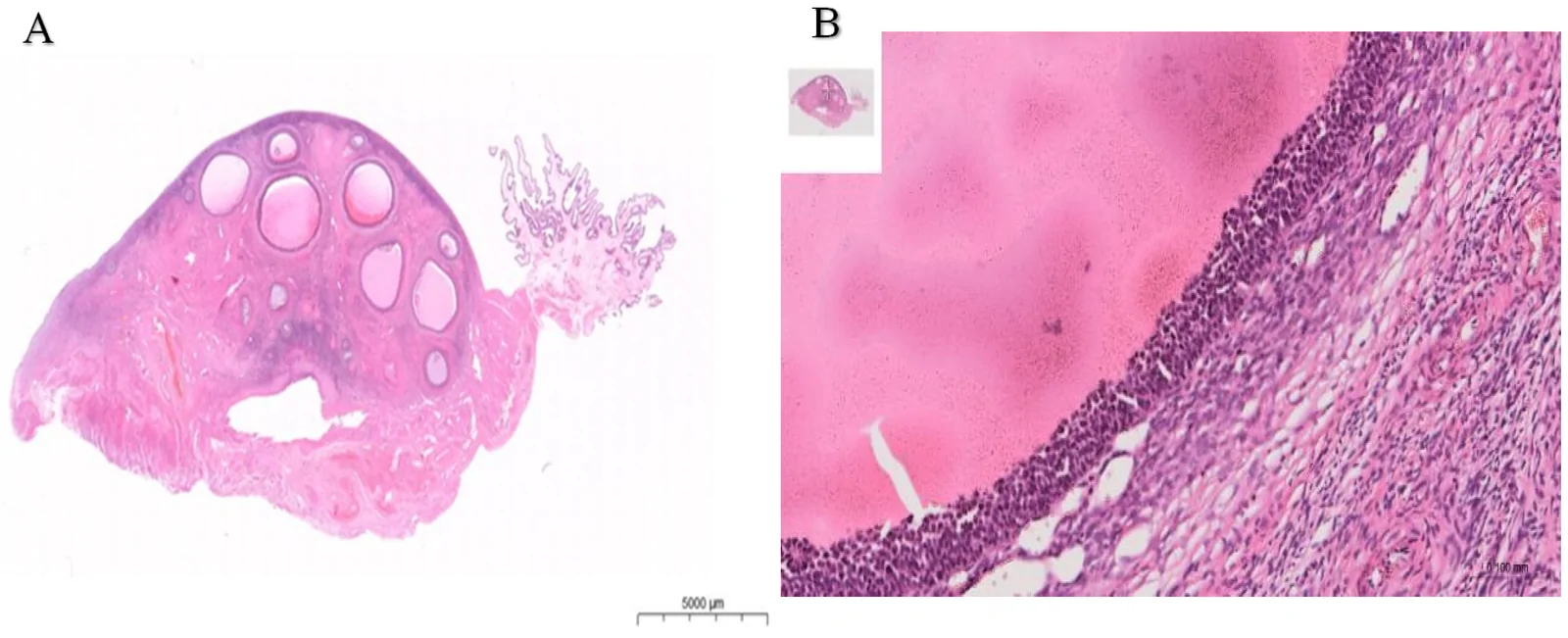
HE-stained paraffin sections of Tibetan sheep ovaries. (**A**) shows the HE-stained paraffin section of ovarian tissue; yellow arrows indicate primary follicles and secondary follicles; black arrows denote Graafian follicles (scale bar = 5000 μm). (**B**) shows a partial magnification of one mature follicle in Figure (**A**) (scale bar = 0.100 mm).

**Figure 4:**
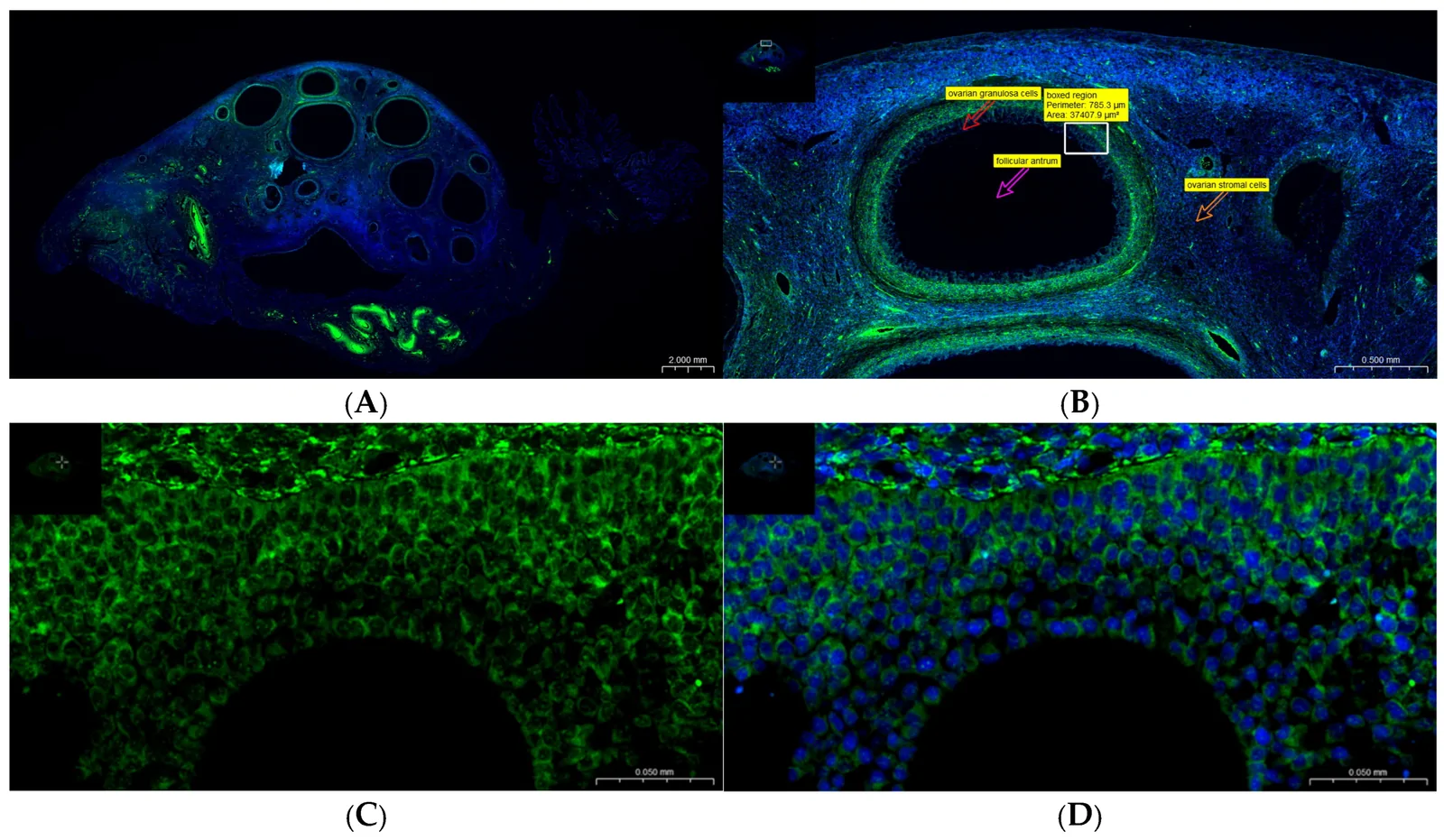
Localization of *GnIH* in ovarian tissues of Tibetan sheep. (**A**) Intact ovarian tissue following in situ hybridization. Green fluorescence shows the localization and expression intensity of the probe, while blue fluorescence corresponds to cell nuclei stained with 4′,6-diamidino-2-phenylindole (DAPI). (**B**) A magnified view of the region in Panel A,Orange arrow, ovarian stromal cells; red arrow, ovarian granulosa cells; purple arrow, follicular antrum; box, magnified region. (**C**) Green fluorescence signals of *GnIH*gene hybridized with probes. (**D**) Merged image of green signals of *GnIH* genes and blue nuclear staining signals.

Ovarian granulosa cells freshly isolated and cultured in vitro from Tibetan sheep exhibited a round morphology under the microscope. Cells began to adhere to the culture plate 5–6 h after seeding and extended pseudopodia to exhibit an irregular triangular shape at 24 h of culture (Figure 2). Shown in Figure 3 and Figure 4 are the ovarian tissues of Tibetan ewes embedded in paraffin and sectioned for fluorescence in situ hybridization with fluorescent probes. Primary follicles, secondary follicles and multiple mature follicles could be observed in ovarian tissues (Figure 3A). The granulosa layer, theca folliculi and stroma were clearly distinguished in the magnified view of a mature follicle (Figure 3B). Green positive hybridization signals of *GnIH* were detected in the ovarian tissues by fluorescence in situ hybridization (Figure 4A). *GnIH* signals were detectable in follicles of different sizes, with enriched signals in the granulosa layer of mature follicles. Under the magnified view, positive signals were mainly localized in follicular granulosa cells, while weak signals were observed in ovarian stromal cells, but no obvious specific signals were present in the follicular antrum (Figure 4B). The individual green-fluorescence channel clearly displayed the distribution pattern of *GnIH* hybridization signals (Figure 4C). The merged image of hybridization signals and DAPI nuclear staining further confirmed that *GnIH*-positive signals were predominantly distributed in the cytoplasm of granulosa cells (Figure 4D).

### 3.3. Effect of Overexpression Plasmid Transfection on GnIH mRNA Expression

**Figure 5:**
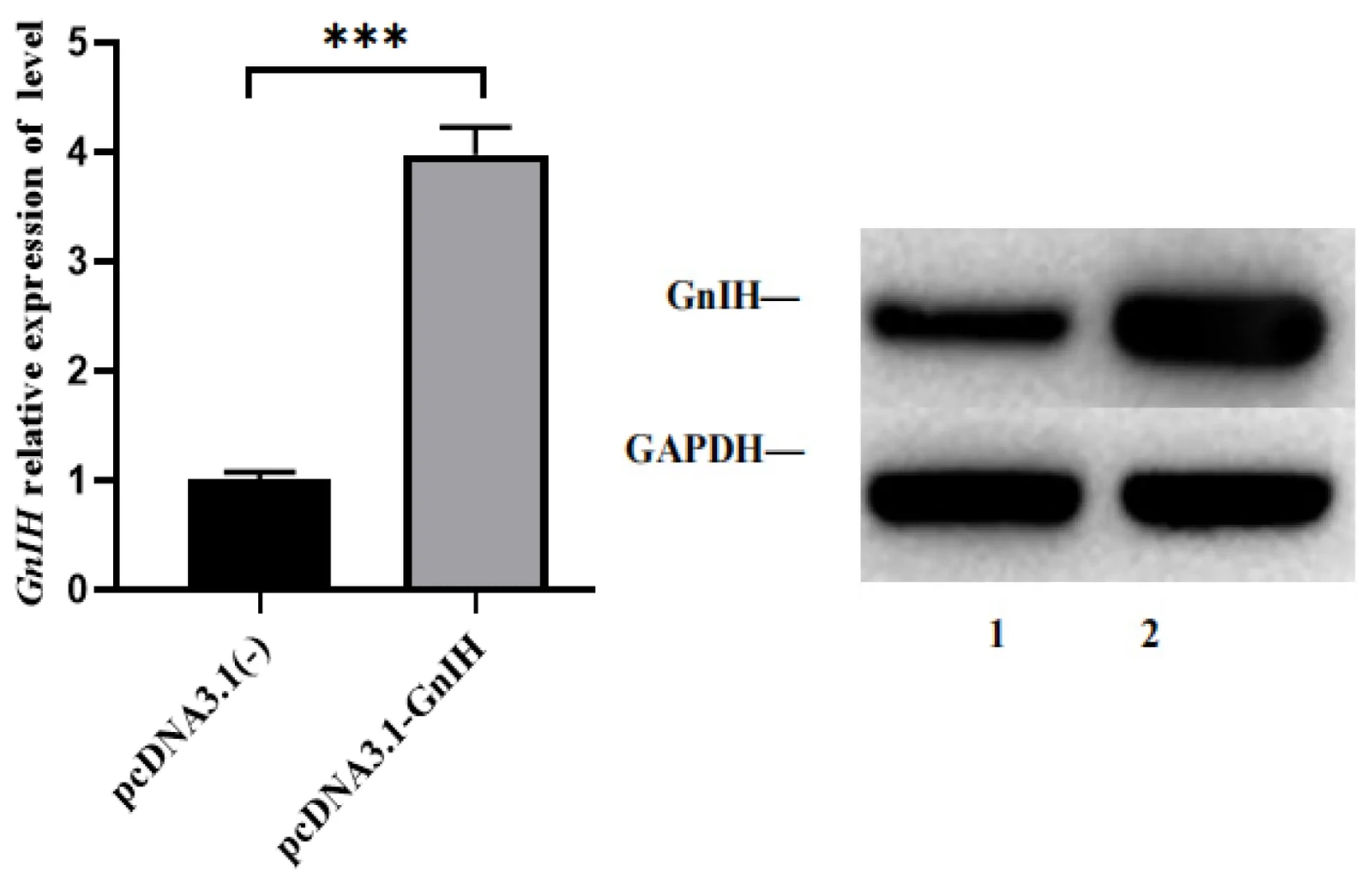
*GnIH* mRNA and protein expression in cells transfected with *GnIH* overexpression plasmid. Lane 1, the pcDNA3.1(-) group, and Lane 2, the pcDNA3.1-GnIH group. Bars represent mean ± SEM (n = 3 biological replicates). *** indicates *p* < 0.001, (Independent Samples *t*-test).

Sheep ovarian granulosa cells transfected with pcDNA3.1-GnIH recombinant plasmid and empty vector pcDNA3.1(-) were, respectively, compared with the empty-vector pcDNA3.1(-) group. It is found that *GnIH* mRNA abundance increased extremely significantly in cells transfected with the recombinant overexpression plasmid (*p* < 0.001) (Figure 5). These results demonstrated that pcDNA3.1-GnIH could efficiently drive *GnIH* expression in Tibetan sheep ovarian granulosa cells. Uncropped western blot images and corresponding grayscale quantification data are provided in Figure S1.

### 3.4. Interference Efficiency Detection of GnIH-Targeted siRNA at mRNA and Protein Levels

**Figure 6:**
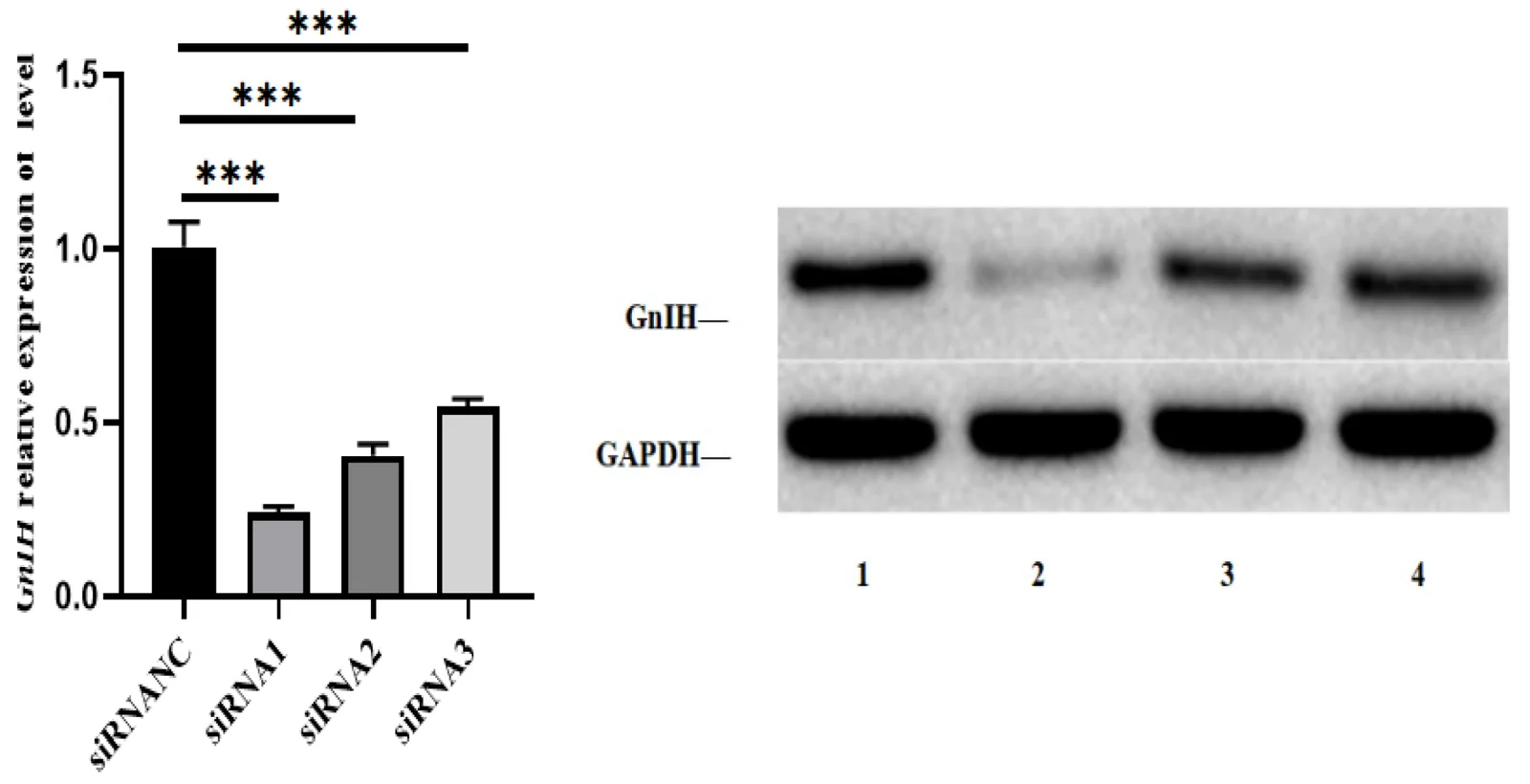
*GnIH* mRNA and protein expression after transfection with interfering small RNAs. Column 1—NC group; Column 2—siRNA1; Column 3—siRNA2; Column 4—siRNA3. Bar length represents mean ± SEM (n = 3 biological replicates). *** indicates *p*< 0.001 (Independent Samples *t*-test).

In this study, three designed siRNAs were separately transfected into Tibetan sheep ovarian granulosa cells. As shown in Figure 6, siRNA1 exhibited the optimal interference efficiency compared with the negative-control siRNA (siRNA-NC) group, with an extremely significant difference (*p* < 0.001) between them. Therefore, siRNA1 was selected for subsequent experiments.

### 3.5. Effect of GnIH on Proliferation of Tibetan Sheep Ovarian Granulosa Cells

**Figure 7:**
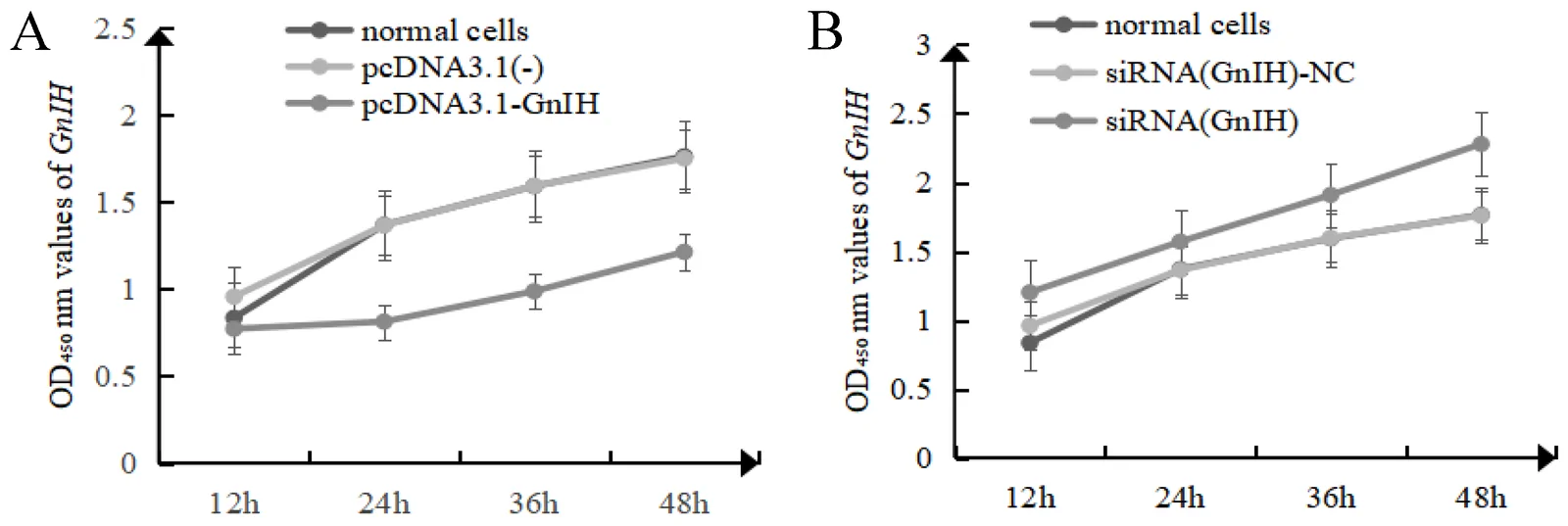
OD_450_ nm values in Tibetan sheep ovarian granulosa cells following *GnIH* overexpression and knockdown. (**A**) OD_450_ nm values of granulosa cells in the normal cells, pcDNA3.1(-) empty-vector control and pcDNA3.1-GnIH overexpression groups at 12 h, 24 h, 36 h and 48 h. (**B**) OD_450_ nm values of granulosa cells in the normal cells, siRNA(GnIH)-NC negative control and siRNA(GnIH) knockdown groups at 12 h, 24 h, 36 h and 48 h.

Ovarian granulosa cells were transfected with overexpression plasmids (pcDNA3.1-GnIH and pcDNA3.1(-)) and interference fragments (siRNA-NC and siRNA-GnIH), respectively. As shown in Figure 7, cell viability increased over time. Compared with the normal cell group and the pcDNA3.1(-) group, the overexpression group (pcDNA3.1-GnIH) significantly reduced the viability of Tibetan sheep ovarian granulosa cells (*p* < 0.01). In contrast, the siRNA-GnIH interference group had markedly elevated cell viability relative to the normal cell group and the siRNA-NC group (*p* < 0.01).

### 3.6. Effects of GnIH Overexpression and Knockdown on Apoptosis of Tibetan Sheep Ovarian Granulosa Cells

**Figure 8:**
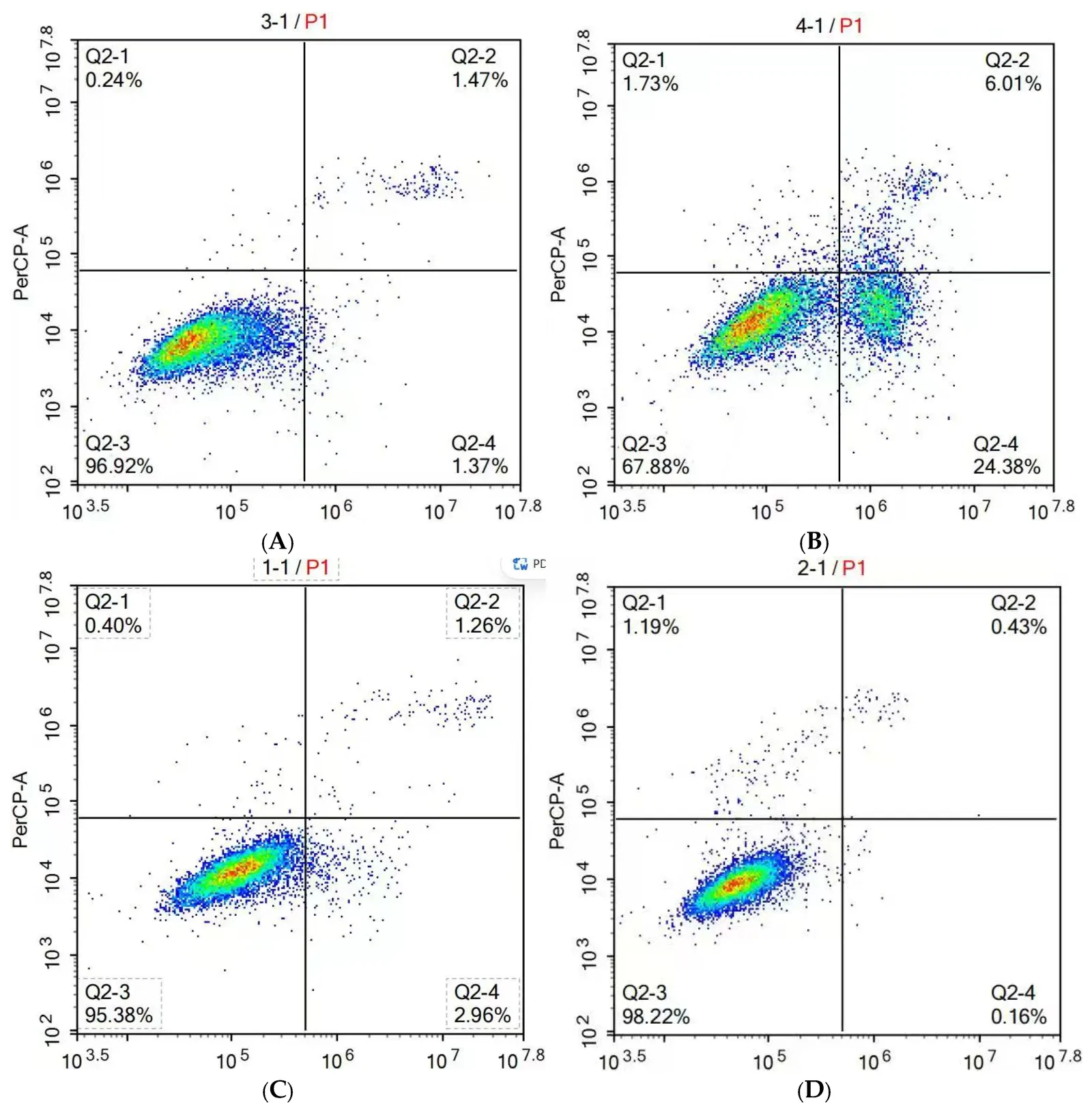
Effects of *GnIH* on apoptosis in Tibetan sheep ovarian granulosa cells: (**A**) pcDNA3.1(-) empty-vector control group; (**B**) pcDNA3.1-GnIH *overexpression* group; (**C**) siRNA(GnIH)-NC negative control group; (**D**) siRNA(GnIH) knockdown group.

Annexin V-FITC/PI double-staining combined with flow cytometry was performed to evaluate the apoptotic level in each group, and the results are presented in Figure 8. Compared with the pcDNA3.1(-) group (Panel A), the pcDNA3.1-GnIH group (Panel B) had a lower proportion of viable granulosa cells (Q2-3 quadrant), whereas its proportions of early-apoptotic cells (Q2-2 quadrant) and late-apoptotic cells (Q2-4 quadrant) were markedly higher. The early- and late-apoptosis rates reached 6.01% and 24.38%, respectively, indicating an elevated total cellular apoptosis level. Conversely, compared with the siRNA-NC group (Panel C), the siRNA-GnIH knockdown group (Panel D) exhibited a higher proportion of viable granulosa cells (Q2-3 quadrant), accompanied by a reduced early-apoptosis rate (0.43%) and late-apoptosis rate (0.16%), and cell apoptosis was markedly suppressed. Collectively, these results suggest that high expression of *GnIH* promotes apoptosis in Tibetan sheep ovarian granulosa cells. Apoptotic rates were quantified based on three independent biological replicates, with statistical comparisons among groups.

### 3.7. Effect of GnIH on the Cycle of Tibetan Sheep Ovarian Granulosa Cells

**Figure 9:**
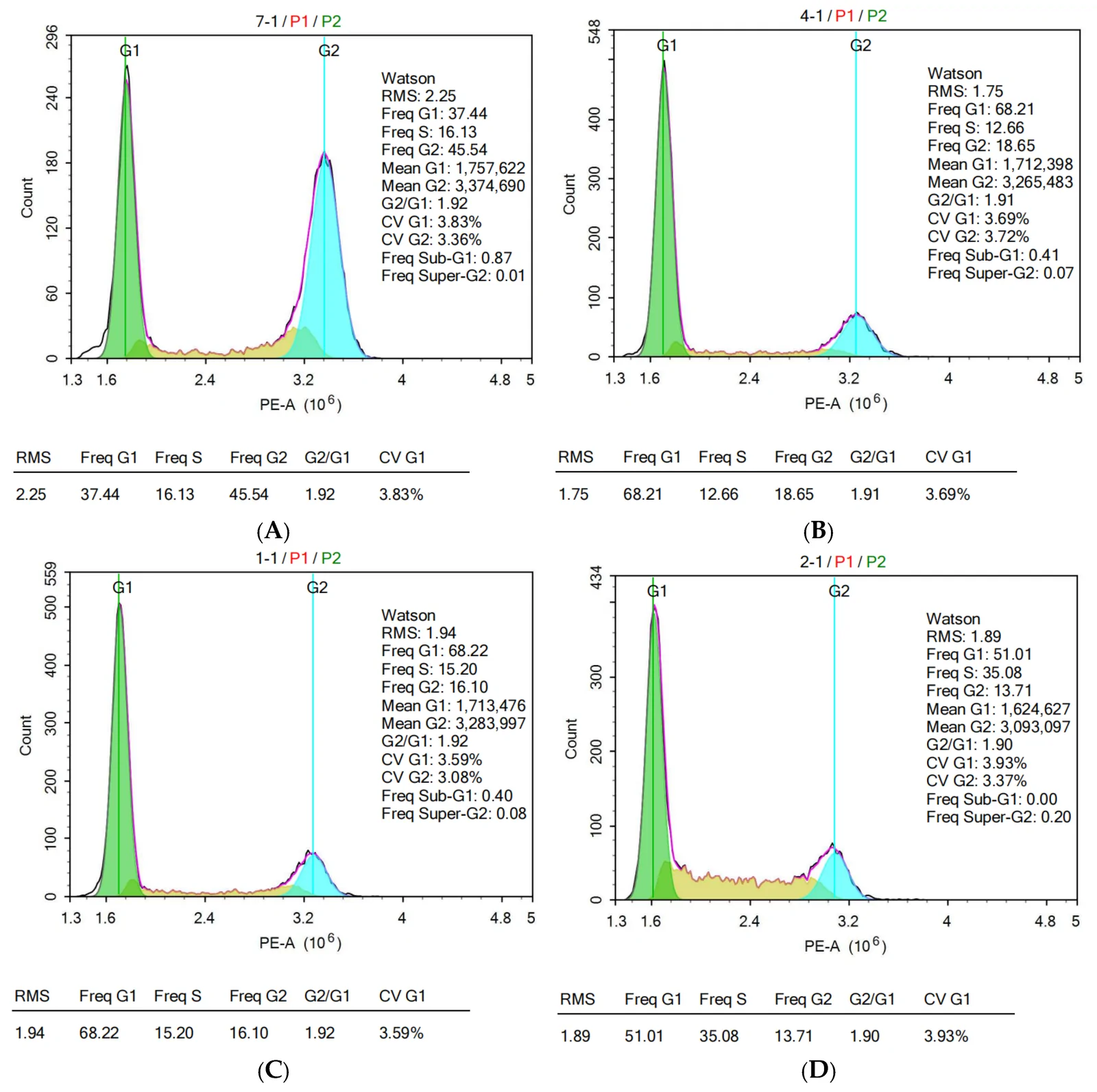
Effects of *GnIH* on cell cycle in Tibetan sheep ovarian granulosa cells: (**A**) pcDNA3.1(-) empty-vector control group; (**B**) pcDNA3.1-GnIH *overexpression* group; (**C**) siRNA(GnIH)-NC negative control group; (**D**) siRNA(GnIH) knockdown group.

The flow cytometry was applied to analyze the cell-cycle distribution. The results of the pcDNA3.1-GnIH overexpression group (Figure 9A,B) showed that the proportions of cells at the G0/G1 phase (pre-DNA-synthesis phase) and S phase (DNA-synthesis phase) were lower in the pcDNA3.1-GnIH group than in the pcDNA3.1(-) group. This finding indicated that *GnIH* overexpression blocked cell-cycle progression and suppressed cell division and proliferation of granulosa cells. By contrast, the results of *GnIH* knockdown (Figure 9C,D) revealed no significant difference in the proportion of G0/G1-phase cells between the siRNA-GnIH group and the siRNA-NC negative-control group. Nevertheless, the proportions of cells in the S phase and G2/M phase (post-DNA-synthesis and mitotic phase) increased after *GnIH* silencing. This suggested that inhibition of *GnIH* expression facilitated the transition of granulosa cells from the DNA-synthesis phase to the mitotic phase, accelerated cell-cycle progression, and consequently enhanced the proliferative capacity of granulosa cells. Quantitative analysis of the proportions of G0/G1, S and G2/M phases was performed based on three independent biological replicates, and statistical comparisons were conducted between groups.

### 3.8. Effects of GnIH on Proliferation- and Apoptosis-Related Genes in Tibetan Sheep Ovarian Granulosa Cells

**Figure 10:**
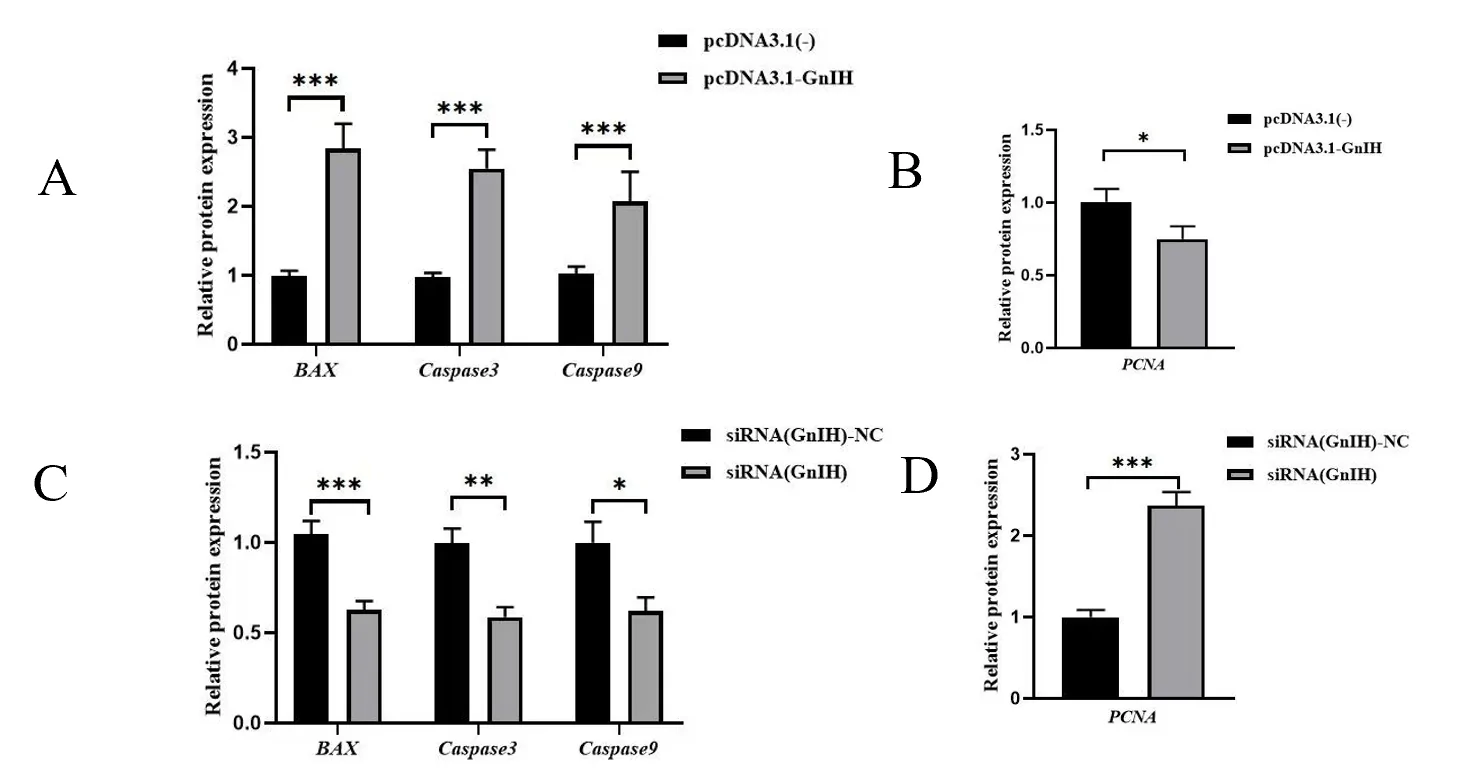
mRNA expression levels of cell proliferation- and apoptosis-related genes after *GnIH* overexpression and knockdown: (**A**) relative mRNA expression levels of apoptosis-related genes after overexpression of *GnIH* genes; (**B**) relative mRNA expression levels of proliferation-related genes after overexpression of *GnIH* genes; (**C**) relative mRNA expression levels of apoptosis-related genes after interference of *GnIH* genes; (**D**) relative *mRNA* expression levels of proliferation-related genes after interference of *GnIH* genes. Bars represent mean ± SEM (n = 3 biological replicates). * indicates *p* < 0.05, ** indicates *p* < 0.01, and *** indicates *p* < 0.001 (Independent Samples *t*-test).

The mRNA expression levels of apoptosis-related genes (*BAX*, *Caspase3*, *Caspase9*) and proliferation-related gene *PCNA* were detected after transfection with overexpression plasmid and GnIH-knockdown siRNA. As illustrated in Figure 10, pcDNA3.1-GnIH significantly upregulated the mRNA levels of pro-apoptotic genes *BAX*, *Caspase3* and *Caspase9* (*p* < 0.001), whereas the mRNA abundance of proliferation-related *PCNA* markedly decreased (*p* < 0.05). In contrast, GnIH silencing via siRNA-GnIH significantly downregulated *BAX* (*p* < 0.001), *Caspase3* (*p* < 0.01) and *Caspase9* (*p* < 0.05), accompanied by a significant increase in *PCNA* mRNA expression (*p* < 0.05).

### 3.9. Effects of GnIH on Steroid Hormone Secretion in Tibetan Sheep Ovarian Granulosa Cells

**Figure 11:**
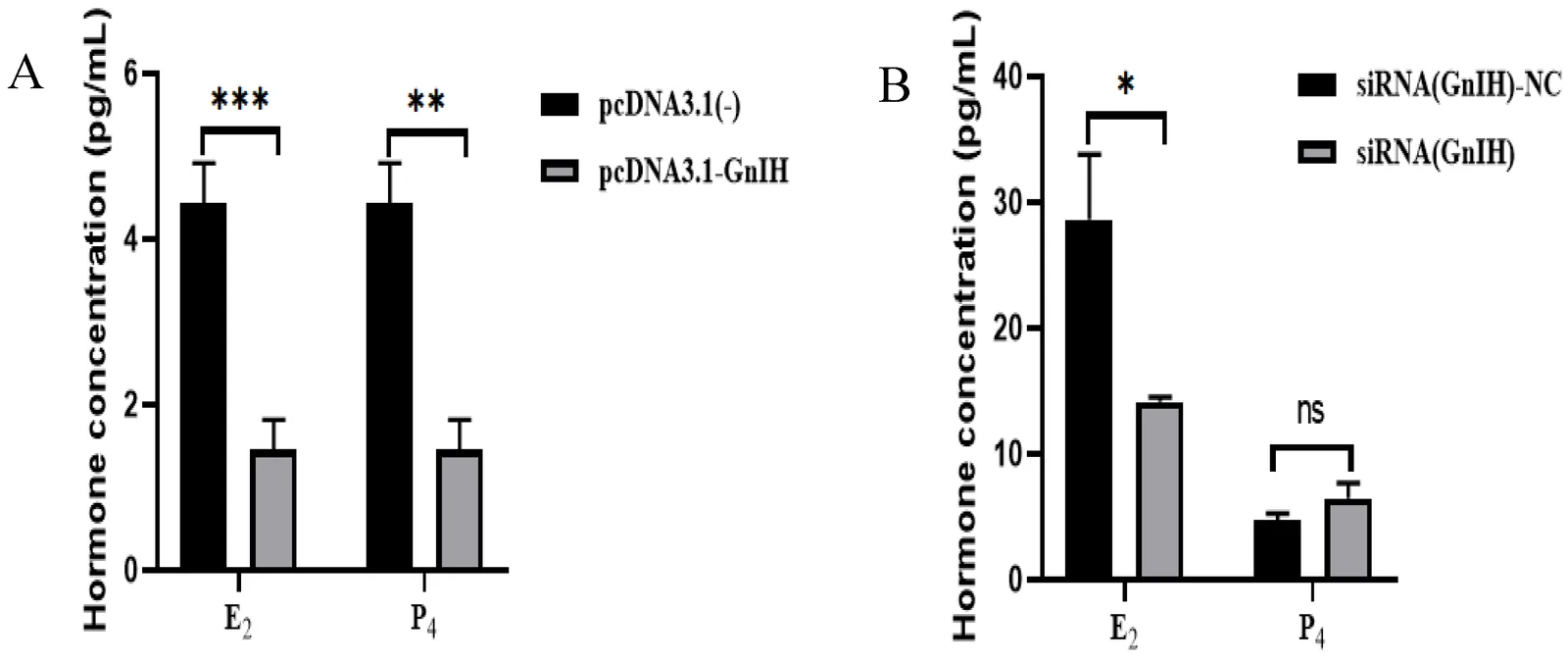
Estrogen and progesterone levels following *GnIH* overexpression and knockdown: (**A**) concentrations of E_2_ and P_4_ after overexpression of *GnIH* genes; (**B**) concentrations of E_2_ and P_4_ after interference of *GnIH* genes. Bars represent mean ± SEM (n = 3 biological *replicates*). ns indicates *p* > 0.05, * *indicates p* < 0.05, ** indicates *p* < 0.01, and *** indicates *p* < 0.001 (Independent Samples *t*-test).

In order to investigate the effect of *GnIH* on steroid hormone synthesis in Tibetan sheep ovarian GCs in vitro, the secretion levels of E_2_ and P_4_ in supernatants were measured by ELISA using commercial E_2_ and P_4_ assay kits. As shown in Figure 11, compared with the pcDNA3.1(-) empty-vector control group, pcDNA3.1-GnIH significantly decreased the secretion of E_2_ (*p* < 0.001) and P_4_ (*p* < 0.01) in Tibetan sheep ovarian granulosa cells. *GnIH* knockdown by siRNA-GnIH significantly elevated E_2_ secretion (*p* < 0.05); although P_4_ secretion exhibited an upward trend, no significant difference was observed (*p* > 0.05). These findings indicated that *GnIH* suppresses E_2_ and P_4_ secretion in sheep ovarian granulosa cells.

## 4. Discussion

Since *GnIH* was first identified in the Japanese quail hypothalamus by Tsutsui’s group in 2000 [19], this neuropeptide has been confirmed as a highly conserved key negative reproductive regulator among vertebrates [20]. It mainly modulates reproductive processes via the hypothalamic–pituitary–gonadal axis and can exert direct effects on gonadal cell development [21]. Multiple studies have demonstrated that *GnIH* was significantly associated with litter size, and its biological effects exhibit obvious concentration dependency: high *GnIH* concentrations downregulate the expression of reproduction- and proliferation-related genes and promote cell apoptosis within 24 h, whereas low concentrations produce the opposite regulatory effects [9,22]. Tissue expression profiling in sheep revealed that *GnIH* was widely distributed across gonadal-axis tissues with a relatively high abundance in the hypothalamus, which was consistent with expression patterns reported in various other animal species [23]. In the present study, mRNA levels of *GnIH* determined in different gonadal-axis tissues of Tibetan sheep were consistent with the findings reported by Ukena et al. [16]. *GnIH* mRNA showed the highest abundance in the hypothalamus, which was significantly higher than that in the ovary, pituitary, uterus and oviduct. The ovary exhibited the second-highest expression level, significantly higher than those of the pituitary, uterus, and oviduct. Recent evidence has indicated that *GnIH* controls final oocyte maturation in zebrafish via paracrine/autocrine manners and cooperates with gonadotropins [1]. Considering the considerable *GnIH* expression detected in the pituitary and ovary in this study, *GnIH* may participate in the regulation of ovarian functions and further influence reproductive performance in Tibetan sheep through both central nervous system and local ovarian pathways.

Follicular development is essential for animal reproductive function. As the core functional cells for folliculogenesis, ovarian granulosa cells activate the major downstream mediator PI3K of follicular development through the KIT/KITL signal, facilitating the transition from primordial follicles to primary follicles [24,25]. Moreover, granulosa cells respond to pituitary-derived FSH to promote follicle maturation and are stimulated by LH to participate in ovulation-related processes in female animals [26,27]. Each primordial follicle consists of a single oocyte surrounded by a monolayer of flattened granulosa cells. As follicles develop into mature follicles, granulosa cells differentiate into mural granulosa cells and cumulus granulosa cells. Mural granulosa cells are responsible for maintaining follicular architecture, whereas cumulus granulosa cells encircle the oocyte [28,29]. These descriptions agree with our fluorescence in situ hybridization results, confirming that *GnIH* is localized in ovarian granulosa cells. *GnIH* has also been detected in theca cells, luteinized granulosa cells, and cellular components of follicular complexes at different follicular developmental stages. Subcellular localization further indicates that *GnIH* may exert critical biological functions in the cytoplasm of granulosa cells. Collectively, these observations suggest that *GnIH* was involved in follicular development in Tibetan sheep.

Granulosa cell proliferation and apoptosis are central drivers determining follicle growth and atresia. Consistent with previous work on porcine granulosa cells [30,31], our functional manipulation of *GnIH* indicates that this peptide restrains granulosa cell survival. Notably, our cell-cycle data reveal a species-specific regulatory pattern: unlike the G2/M arrest reported in porcine granulosa cells [30], *GnIH* overexpression in Tibetan sheep granulosa cells reduced the proportions of G0/G1 and S-phase cells, while *GnIH* knockdown shifted cell distribution toward S and G2/M phases. This divergence implies that *GnIH* does not act on a universally conserved cell-cycle checkpoint across ruminants, and its cell-cycle regulatory network is shaped by species-specific ovarian backgrounds. The transcriptional responses of *BAX*, *Caspase 3*, *Caspase 9* and *PCNA* further support the pro-apoptotic and anti-proliferative roles of *GnIH*, which align with observations in mice, yaks and Tan sheep [32,33]. Consistent with our results in Tibetan sheep granulosa cells, a recent study in Tan sheep demonstrated that *GnIH* suppresses granulosa cell proliferation, triggers apoptosis and reduces estradiol and progesterone synthesis through PI3K/AKT/mTOR and MAPK/ERK1/2 signaling cascades [34]. These parallel observations support the conserved inhibitory role of this peptide on ovine granulosa cell function. However, Tibetan sheep are adapted to high-altitude hypoxic environments, which may shape distinct endocrine regulatory patterns compared with lowland Tan sheep. Further work is required to clarify whether altitude-associated variation modifies the downstream signaling intensity of *GnIH* in ovine follicles. Although the PI3K/AKT/mTOR cascade has been proposed as a downstream route for *GnIH* in other models [34], this pathway remains to be validated in Tibetan sheep granulosa cells. It should be noted that the CCK-8 assay assesses overall cell viability instead of cell proliferation exclusively. The decreased viability observed after GnIH treatment may be partly attributed to elevated apoptosis, which is consistent with our apoptosis and cell-cycle results.

As core steroid hormones synthesized by granulosa cells, estradiol and progesterone can in turn modulate granulosa-cell proliferation and apoptosis in an autocrine manner [35,36,37]. Physiological concentrations of estradiol activate the PI3K-AKT signaling cascade, upregulate proliferative-related genes such as *PCNA*, increase the abundance of anti-apoptotic Bcl-2 protein, sustain granulosa-cell survival, and prevent follicular atresia [38,39]. The biological actions of progesterone on granulosa cells depend on cellular differentiation status. Prior to follicular luteinization, progesterone generally suppresses proliferation and induces apoptosis. After LH-surge-triggered luteinization of granulosa cells, progesterone shifts its function toward maintaining luteal-cell viability [40,41]. Our present results revealed that *GnIH* alters estradiol and progesterone secretion in granulosa cells. This creates a plausible dual regulatory model: *GnIH* may directly tune apoptosis and proliferation gene expression while indirectly reshaping cell fate by modifying intrafollicular steroid signaling. Whether steroid changes are a cause or a secondary consequence of *GnIH*-induced cell-cycle arrest and apoptosis cannot be fully resolved by the present transfection model.

This study has several important limitations that should be considered when interpreting the conclusions. First, the number of biological replicates in our in vitro granulosa cell experiments was relatively small, which may limit statistical robustness and reduce the capacity to capture biological variation among individual samples. Second, the reproductive stage and estrous cycle status of Tibetan sheep strongly shape granulosa cell endocrine phenotypes, apoptosis susceptibility and steroidogenic capacity. Since granulosa cells were collected without strict stratification by reproductive phase, we cannot exclude the potential confounding effects of different ovarian physiological states on our results. In addition, all conclusions are derived from an in vitro cultured cell system, which cannot recapitulate the complex endocrine microenvironment within the ovarian follicle in vivo. Therefore, the regulatory effects of *GnIH* reported here should be extrapolated cautiously. Future studies with expanded biological replicates, carefully staged ovarian samples and in vivo intervention trials are required to confirm the reproductive function of *GnIH* in Tibetan sheep. Another limitation of the present study is the relatively small number of biological replicates (n = 3), which is a common practical constraint for experiments using primary Tibetan sheep granulosa cells owing to the difficulty of isolating sufficient high-quality cells. Future studies with increased sample sizes will help to confirm our observations.

## 5. Conclusions

This study systematically explored the expression patterns of *GnIH* genes in gonadal-axis tissues and ovarian granulosa cells of Tibetan sheep. The results demonstrated that *GnIH* was expressed in the hypothalamus, pituitary, ovary, uterus and oviduct. The highest mRNA abundance of *GnIH* was observed in the hypothalamus, followed by the ovary. Subcellular localization showed that *GnIH* was mainly distributed in the cytoplasm of ovarian granulosa cells, suggesting that *GnIH* participates in the regulation of ovarian physiological activities in Tibetan sheep through both central regulatory pathways and local ovarian mechanisms.

Cell functional experiments verified that *GnIH* exerts notable regulatory functions on proliferation, cell-cycle progression, apoptosis and hormone secretion of Tibetan sheep ovarian granulosa cells. Upregulating *GnIH* expression significantly impaired granulosa cell viability, blocked cell-cycle progression, increased the expression of apoptosis-related genes and suppressed cell proliferation while decreasing the synthesis and secretion of estradiol and progesterone. On the contrary, endogenous *GnIH* interference effectively improved the proliferative potential of granulosa cells, alleviated cell apoptosis, accelerated cell-cycle progression and increased estradiol secretion.

## Figures and Tables

**Table 1:** *GnIH* siRNA sequences.

Items	Target Position (nt)	Sense (5′–3′)	Antisense (5′–3′)
siRNA (GnIH)-NC		GAAGAAGAAAGGAGCACUATT	UAGUGCUCCUUUCUUCUUCTT
siRNA (GnIH)-1	289–309	UCACCAAGACCCUGAGUAATT	UUACUCAGGGUCUUGGUGATT
siRNA (GnIH)-2	419–439	GAAAAUAGAUGAUGCAGAATT	UUCUGCAUCAUCUAUUUUCTT
siRNA (GnIH)-3	555–575	UUCUCCGAACGUGUCACGUTT	ACGUGACACGUUCGGAGAATT

**Table 2:** Primer information of RT-qPCR.

Gene		Primer Sequence (5′→3′)	Size (bp)
*GnIH*	F	TCAAAGGAAAGAGAAGAGGAAAAAAC	104
	R	CATCCAGTGCATAACAATCAGAGT	
*PCNA*	F	AGTGGCGTGAACCTACAGAG	86
	R	CGGTAAGTGTCGAAGCCCTC	
*BAX*	F	ACAGGGGCCCTTTTGCTTC	127
	R	TCAGACACTCGCTCAGCTTC	
*Caspase3*	F	CAGACGTGGATGCAGCAAAC	207
	R	GATCGACGGGTCCATTGGTT	
*Caspase9*	F	TCTCCTGGAGGGATCCCAAG	118
	R	ACAGCATTAGCGACCCTGAG	
*β-actin*	F	ACGGGCAGGTCATCACCATCGG	159
	R	TGTTGGCGTAGAGGTCCTTGCGG	

## Data Availability

The data that support the findings are unavailable for public release at present due to the ongoing project research.

## Supplementary Materials

The following supporting information can be downloaded at: https://www.mdpi.com/article/10.3390/ani16213517/s1, Figure S1. Original uncropped western blot membranes of GnIH and GAPDH in Tibetan sheep ovarian granulosa cells.Original uncropped western blot membranes for GnIH and GAPDH from three biological replicates of Tibetan sheep ovarian granulosa cells. Lane order: siRNA(GnIH)-NC, siRNA(GnIH)-1, siRNA(GnIH)-2, siRNA(GnIH)-3, pcDNA3.1(-) and pcDNA3.1-GnIH. The corresponding gray values and intensity ratios (GnIH/GAPDH) for each replicate are listed in the table below the membranes.
