# Supplementary material for: GnIH Modulates Proliferation, Apoptosis, and Steroid Hormone Secretion in Tibetan Sheep Granulosa Cells
Source: Animals (Basel). 2026 Nov 6;16(21):3517. doi: 10.3390/ani16213517 (PMC13761438; doi:10.3390/ani16213517)
Supplement: Supplementary file 1 [file animals-16-03517-s001.zip › animals-4622514-supplementary.pdf]

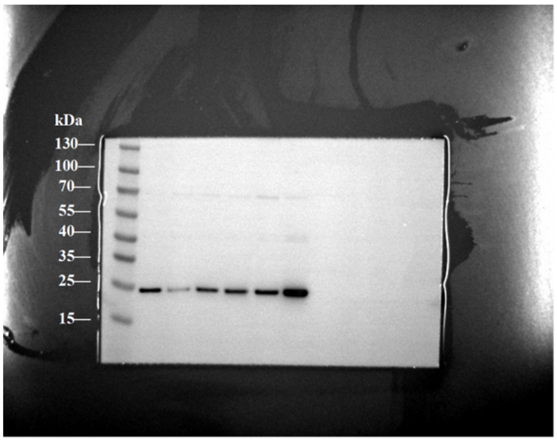
kDa
130
100
70
55
40
35
25
15

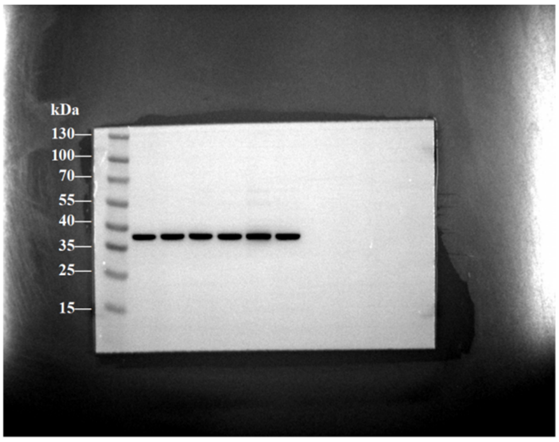
kDa
130
100
70
55
40
35
25
15

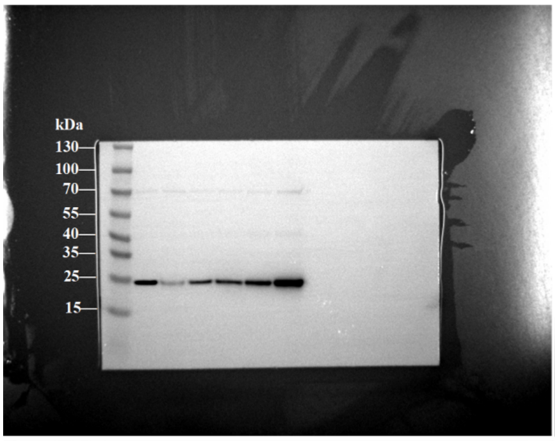
kDa
130
100
70
55
40
35
25
15

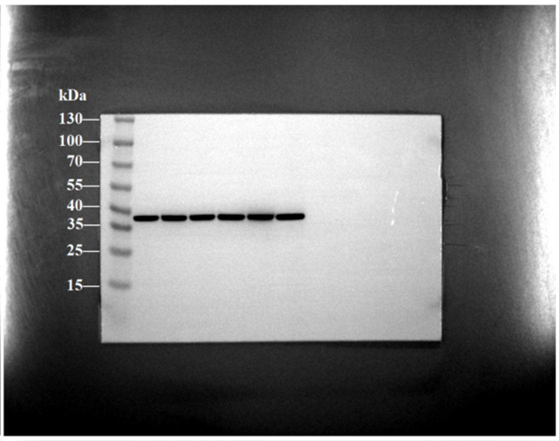
kDa
130
100
70
55
40
35
25
15

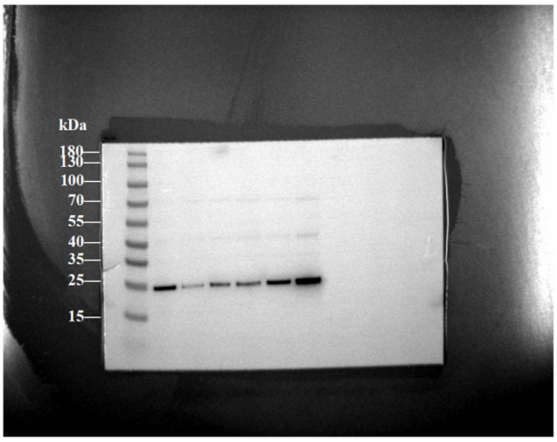
kDa
180
130
100
70
55
40
35
25
15

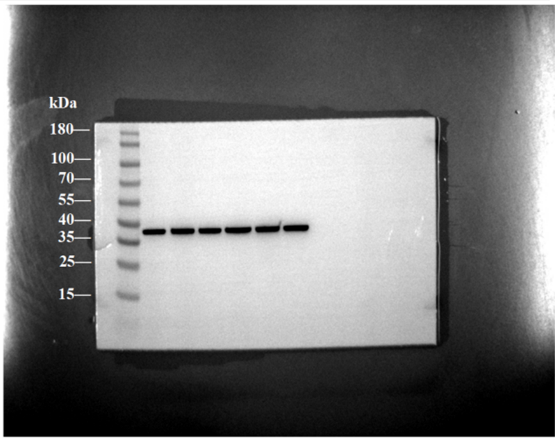
kDa
180
100
70
55
40
35
25
15

Table: Grayscale values and relative expression ratios of GnIH protein detected by Western blot. Three biological replicates were set for each treatment group. GAPDH served as the loading control

replicate	gray value	siRNA (GnIH) -NC	siRNA (GnIH) -1	siRNA (GnIH) -2	siRNA (GnIH) -3	pcDNA3.1 (-)	pcDNA3.1-GnIH
1	GnIH	453.29	114.47	271.22	293.41	457.28	1828.31
	GAPDH	1966.50	1966.03	1940.03	1969.33	1959.82	1977.85
2	GnIH	511.05	128.62	308.27	319.93	501.93	2055.31
	GAPDH	1840.78	1883.99	1830.67	1831.16	1850.89	1857.51
3	GnIH	460.18	118.01	277.80	294.23	466.36	1901.56
	GAPDH	1899.03	1933.79	1899.91	1897.63	1889.77	1926.39
1	intensity ratios	0.23	0.06	0.14	0.15	0.23	0.92
2		0.28	0.07	0.17	0.17	0.27	1.11
3		0.24	0.06	0.15	0.16	0.25	0.99

Figure S1. Western blot analysis of GnIH protein expression in Tibetan sheep ovarian granulosa cells after transfection. The left panels show the bands of GnIH protein, and the right panels represent GAPDH as the loading control. Three independent biological replicates were performed.
